# Supplementary material for: Epithelial Regeneration Ability of Crohn’s Disease Assessed Using Patient-Derived Intestinal Organoids
Source: Int J Mol Sci. 2021 Jun 2;22(11):6013. doi: 10.3390/ijms22116013 (PMC8199630; doi:10.3390/ijms22116013)

Supplementary Material

Supplementary Figure S1. The response of intestinal organoid according to TNFα concentration in culture medium.

(**A**) Morphological changes in control jejunal organoids with incremental increases in the TNFα concentration (n = 2).

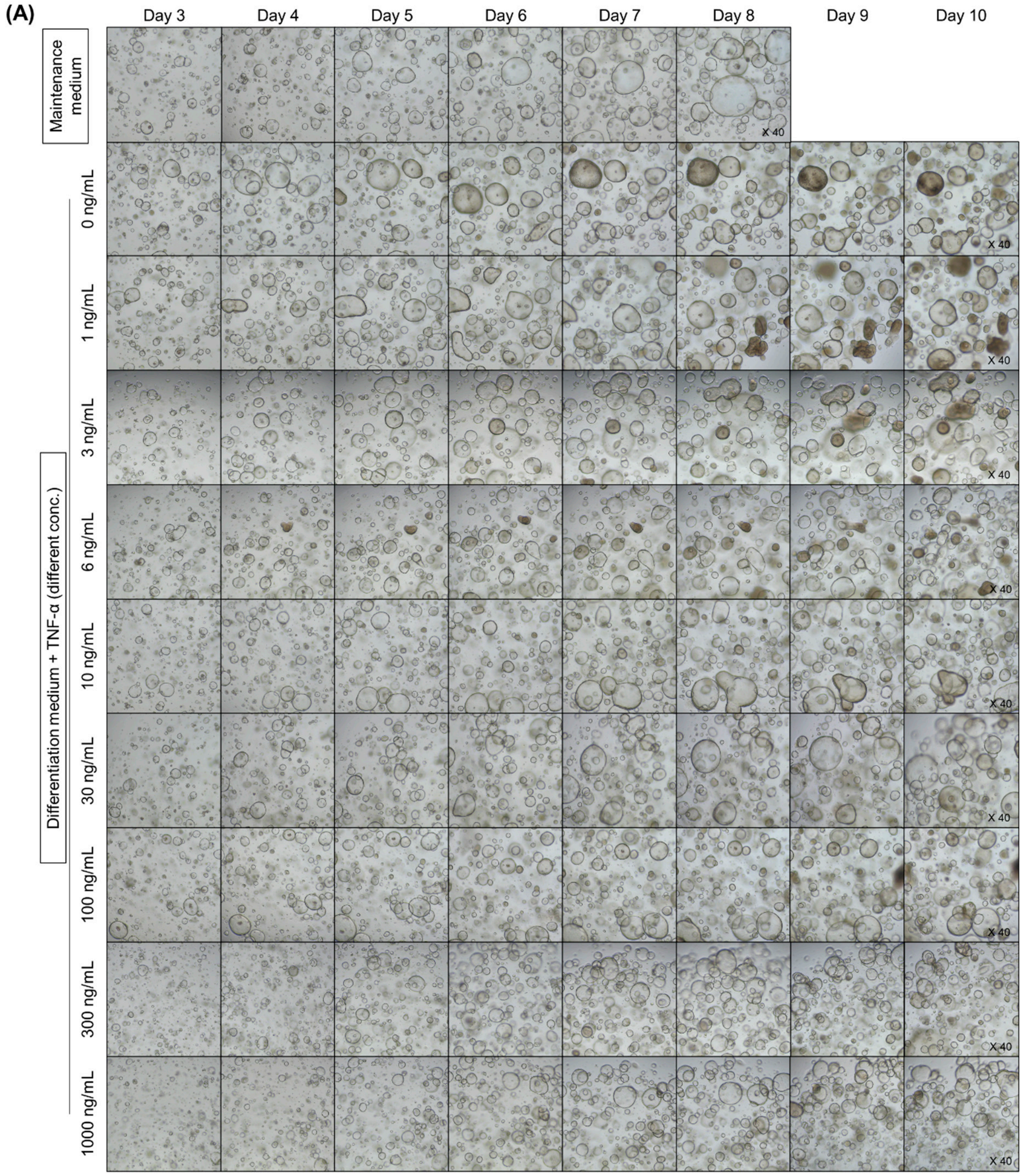

(**B**) Morphological changes in ileal organoids with incremental increases in the TNFα concentration (n = 2).

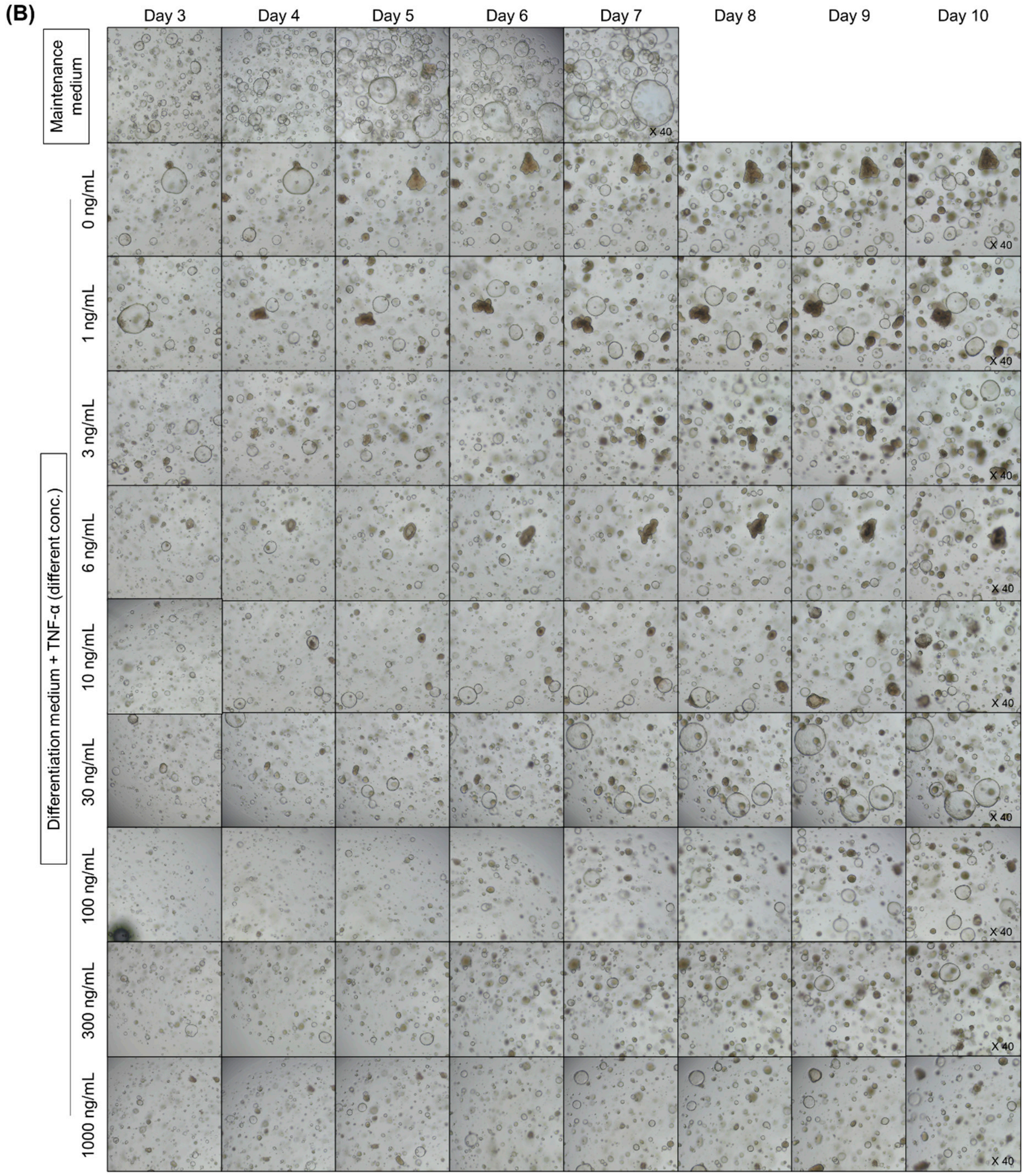

(**C**) MTT assay for organoid viability (n = 4).

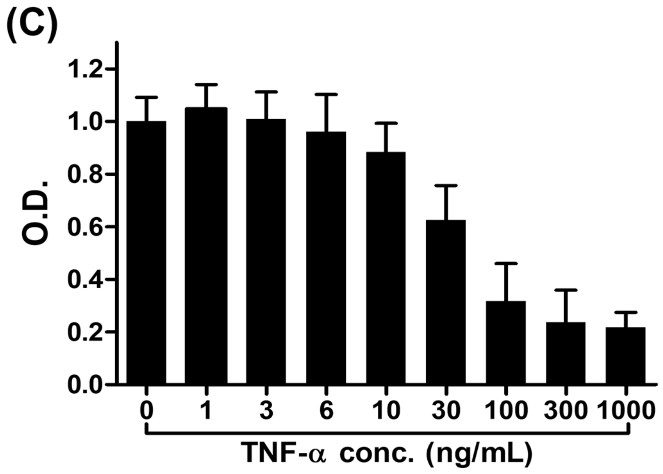

(**D**) Enteroid/spheroid ratio of human intestinal organoids (n = 4).

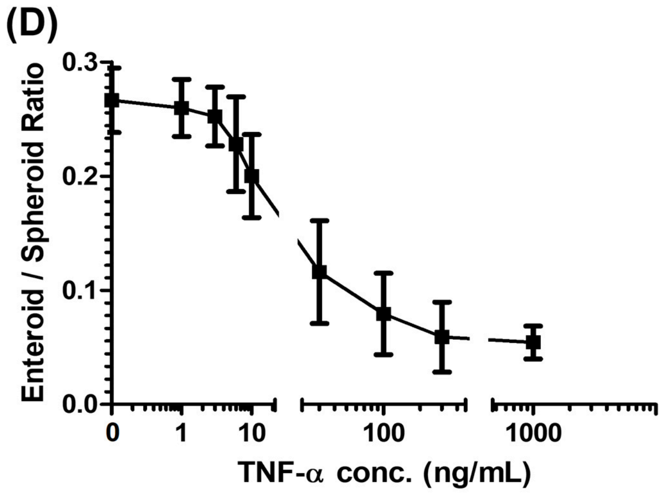

Supplementary Figure S2. Distribution of EdU+ cells 2, 4, 6, 12, and 24 h after EdU administration in control enteroids

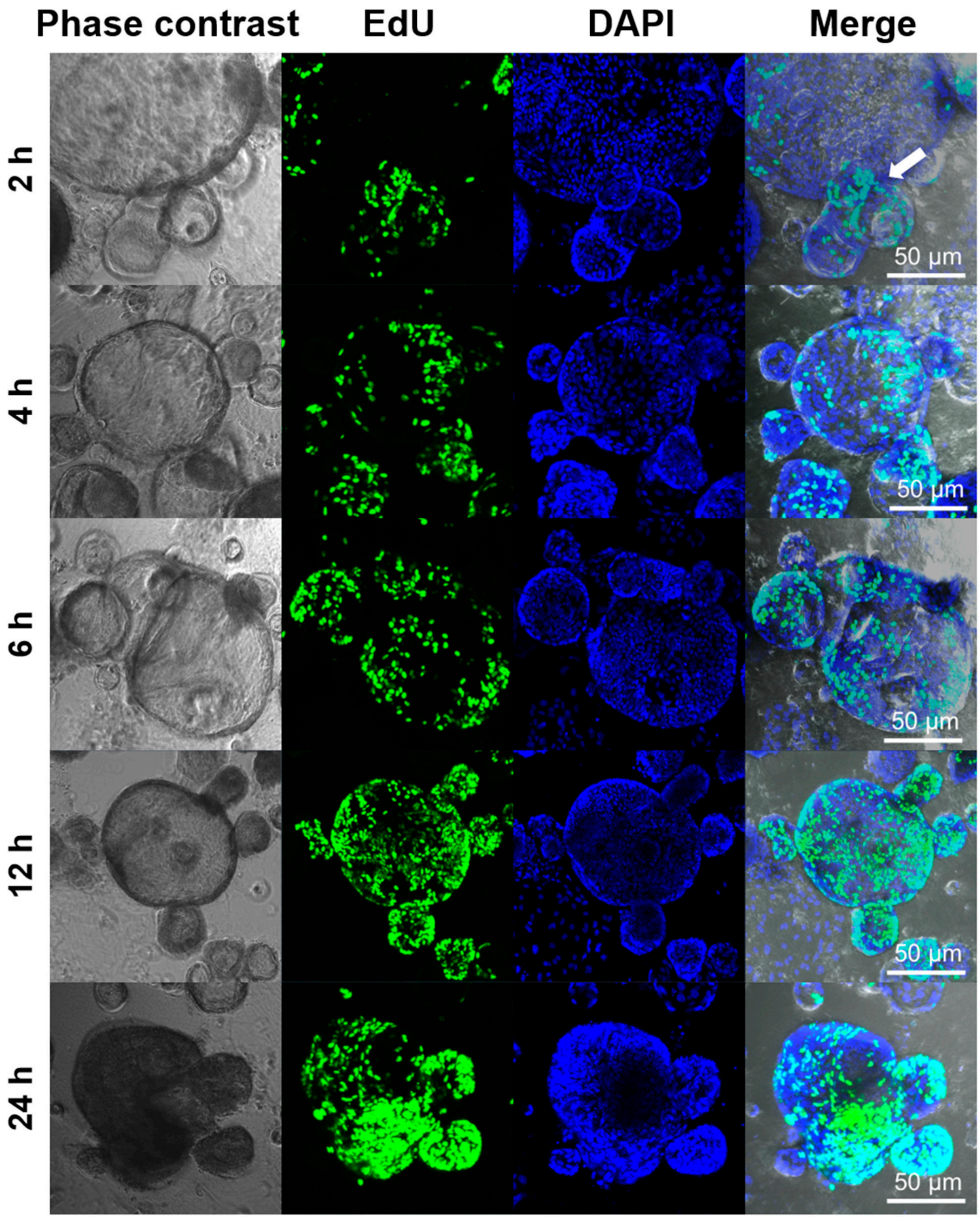

Supplement: Supplementary file 1 [file ijms-22-06013-s001.zip › ijms-1238782-SI.pdf]
